# Delayed ankle muscle reaction time in female amateur footballers after the first 15 min of a simulated prolonged football protocol

**DOI:** 10.1186/s40634-020-00275-1

**Published:** 2020-07-25

**Authors:** Daniel T. P. Fong, Wing-Ching Leung, Kam-Ming Mok, Patrick S. H. Yung

**Affiliations:** 1 National Centre for Sport and Exercise Medicine, School of Sport, Exercise and Health Sciences, Loughborough University, Loughborough, UK; 2 Department of Orthopaedics and Traumatology, Prince of Wales Hospital, Faculty of Medicine, The Chinese University of Hong Kong, Hong Kong, China; 3 Sports Medicine Centre, Elite Training Science & Technology Division, Hong Kong Sports Institute, Hong Kong, China; 4 Student Services Centre, Lingnan University, Hong Kong, China

**Keywords:** Ankle sprain, Ligamentous sprain, Syndesmotic injury, Soccer, Sports medicine, Ankle injuries, Biomechanics

## Abstract

**Purpose:**

Ankle sprain injury rate is reported to be higher towards the end of a football match. Muscle fatigue may contribute to the delayed muscle reaction and subsequent injury. This study investigated the ankle muscle reaction time during a simulated, prolonged football protocol.

**Methods:**

Seven amateur female football players participated in a 105-min simulated, prolonged football protocol. An ankle muscle reaction test was conducted with a pair of ankle sprain simulators at a scheduled interval every 15-min. The reaction times of peroneus longus, tibialis anterior, and lateral gastrocnemius were collected using an electromyography system sampling at 1000 Hz. Repeated measures one-way multivariate analysis of variance with post-hoc paired t-tests were conducted to evaluate if the reaction time at each time point significantly differed from baseline. Statistical significance was set at *p* < 0.05 level.

**Results:**

Reaction times started from 40.5–47.7 ms at baseline and increased to 48.6–55.7 ms at the end. Reaction times significantly increased in all muscles after the first 15 min except for the dominant lateral gastrocnemius. Increased reaction times were seen in the non-dominant limb after 60 min for tibialis anterior, after 75 min for peroneus longus, and after 90 min for the lateral gastrocnemius.

**Conclusions:**

Delayed reaction time of the ankle muscles were found after the first 15 min and in the final 45 min of a simulated prolonged football protocol. Strategies for injury prevention should also focus on tackling the delayed ankle muscle reaction time in the acute phase (the first 15 min), in addition to the latter minutes in the second half.

**Level of evidence:**

Controlled laboratory study, Level V.

## Background

World-wide participation rates in women’s football increased dramatically from 2010 to 2020 and are projected to double by 2026 [1]. Historically, injury incidence has been high in women’s football [2] and remains so today [3]. It is expected that female football-related injuries and longer-term wellbeing will become a significant topic in sports medicine as evidenced by several recent publications [4–6]. A recent systematic review also suggested that recreational football practice is medicine – it is beneficial for cardiovascular and bone health, body composition, type 2 diabetes, and prostate cancer [7]. Previous research has demonstrated that greater attention to injury prevention can mitigate injury risk, allowing athletes to fully benefit from football practice. In this scenario, additional gaps need to be fulfilled regarding excessive ankle inversion caused by incorrect landing posture [8], muscle fatigue during prolonged football exercise [9] and delayed peroneal muscle reaction time [10]. If we can understand the cause of injuries better and introduce appropriate injury prevention strategies, we can ensure that footballers enjoy the health benefits of the game rather than suffer negative consequences.

Systematic reviews [11, 12] and also some recent epidemiology studies on female footballers [13, 14] suggest that ankle sprain is the most common single type of injury in football. In women’s football, an epidemiological study on German premier league players reported that ankle sprains accounted for 35.4% of all injuries incurred in one season [15]. The commonly suggested aetiology of inversion type ankle sprain injury is incorrect positioning of the foot when transitioning from a non-weight-bearing to weight-bearing situation. An aberrant plantar flexed position of the ankle joint [8] increases the torque around the sub-talar joint thus increasing the likelihood of an inversion sprain. Peak inversion during ankle sprain has been reported to reach between 48 and 78 degrees [16, 17], with other studies reporting aberrant inversion being a common finding in chronic ankle instability [18, 19]. The delayed reaction time of the peroneal muscles at the lateral aspect of the ankle, which is usually 60–90 ms, is perhaps too slow to catch up with a quick inversion type ankle sprain motion that happens within 50 ms [16]. Previous studies also suggested that in real football game situations, ankle sprains are more likely to occur during the latter minutes of the first half and during the second half [20]. A recent review suggested that muscle fatigue by the end of a prolonged football game may contribute to the decline in the capability of muscle to generate force, the fall in physical performance, and reduced central drive from the nervous system. All these may predispose the athlete to ankle sprain injury [9]. Another recent study also suggested that football simulated fatigue resulted in balance impairment, which may be a contributory factor toward increased injury risk in the latter part of football games [21].

The presence of fatigue and chronic ankle instability together could disrupt dynamic postural control of the ankle joint [22], making the ankle joint more vulnerable to sustain an ankle sprain injury. To simulate the conditions experienced with prolonged football exercise, there have been numerous studies that have fatigued the ankle evertor muscles via repetitive exercise performed on an isokinetic dynamometer [23]. However, these protocols may not truly represent the physical demands of gameplay and the multi-directional nature of the sport [24]. We aimed to study the ankle evertor muscle fatigue in a realistic simulation of a football match to better understand the aetiology of ankle sprain injury. The purpose of this study was to investigate the ankle muscle reaction in a group of female amateur football players during a functional prolonged football exercise protocol. The hypothesis was that ankle evertor muscle fatigue will cause an increase in the reaction time of the lower leg muscles to an ankle inversion perturbation.

## Methods

### Participants

Seven female football players from the local female amateur football league participated in this study. The exclusion criteria included ankle instability, balance problems, serious foot problems, or lower limb or back fracture within 1 year, as evaluated by an orthopaedic specialist. The subjects were instructed not to perform any vigorous exercise 24 h before testing. Informed consent was obtained from each subject. The Joint Chinese University of Hong Kong – New Territories East Cluster Clinical Research Ethics Committee approved the study (CRE-2010.587).

### Sample size calculation

Sample size estimation was done in G*Power software (Germany), based on a previous study, which reported that the reaction time of peroneus longus to be 68.8 ± 4.5 ms in the stable ankle, and 84.5 ± 4.6 ms in the unstable ankle without tape [25]. By setting the level of significance to 0.05 and the statistical power to 0.80 in a two-tailed test on two independent groups, the estimated required sample size was calculated to be 6.

### Experimental design

**Fig. 1:**
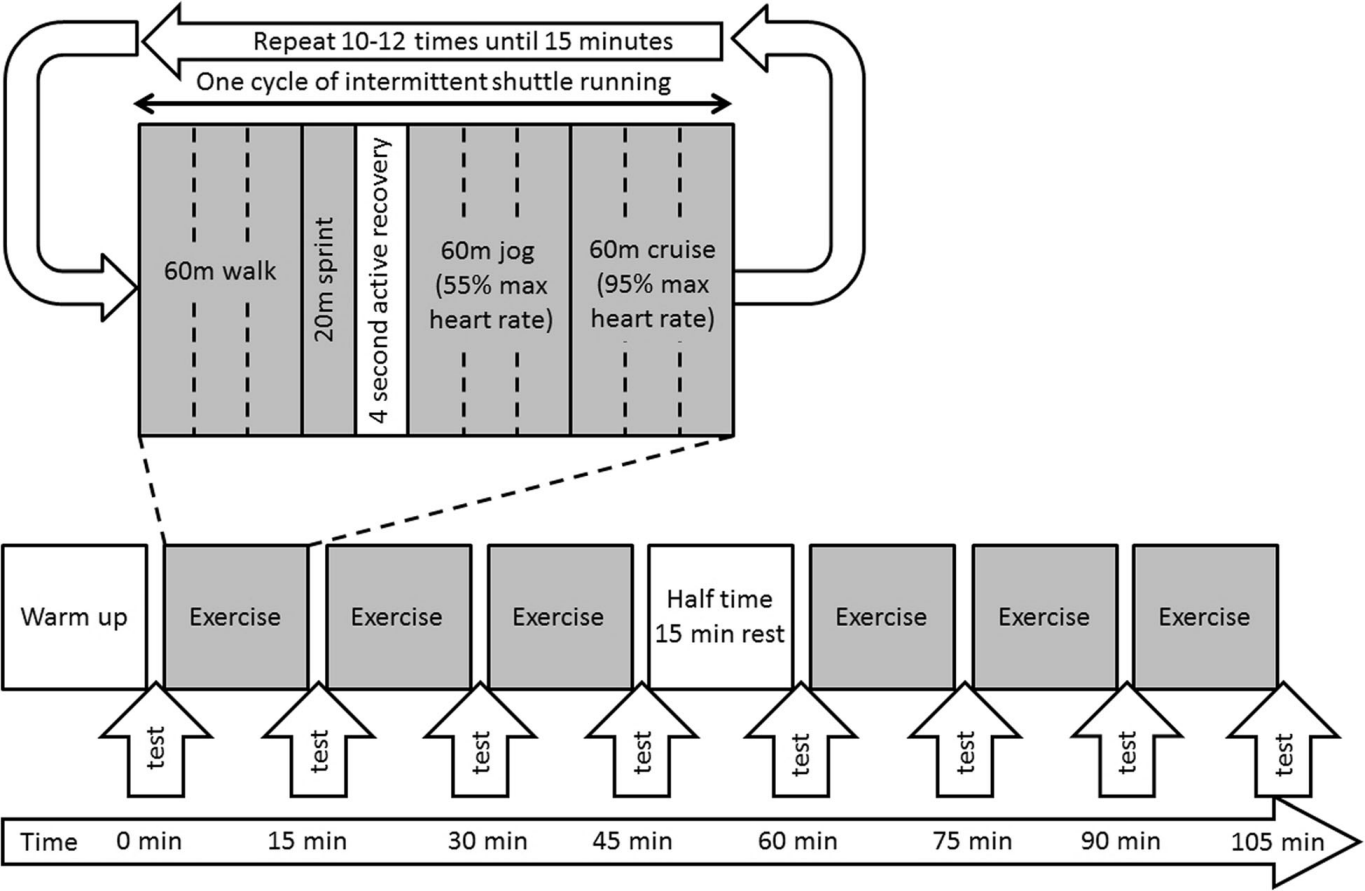
The prolonged simulated football protocol used in this study

Each subject participated in a protocol (Fig. 1) modified from the Loughborough Intermittent Shuttle Test [26]. Before the exercise protocol, a standardized warm-up consisting of jogging and stretching was performed for 15 min. All subjects were equipped with a heart rate monitor (Polar Sport tester, Polar Electro Oy, Kempele, Finland), and their maximum heart rate was estimated by 220 minus their age. The protocol contained two sessions of 45-min continuous intermittent shuttle running exercises on hard ground, with a 15-min rest in between. The participants were required to run between two cones 20 m apart, at speeds related to the estimated individual heart rate values. Each cycle of the intermittent shuttle running exercise consisted of 60 m walking, 20 m sprinting, 4-s recovery walks, 60 m jogging at 55% maximum heart rate, and 60 m cruising at 95% maximum heart rate. A research assistant monitored the heart rate and gave verbal instructions to the subject to run faster or slower to achieve the targeted heart rate. The cycle was repeated for around 10–12 times until 15 min were reached, at which point a short interval was taken to conduct an ankle muscle reaction test before the next cycle began.

**Fig. 2:**
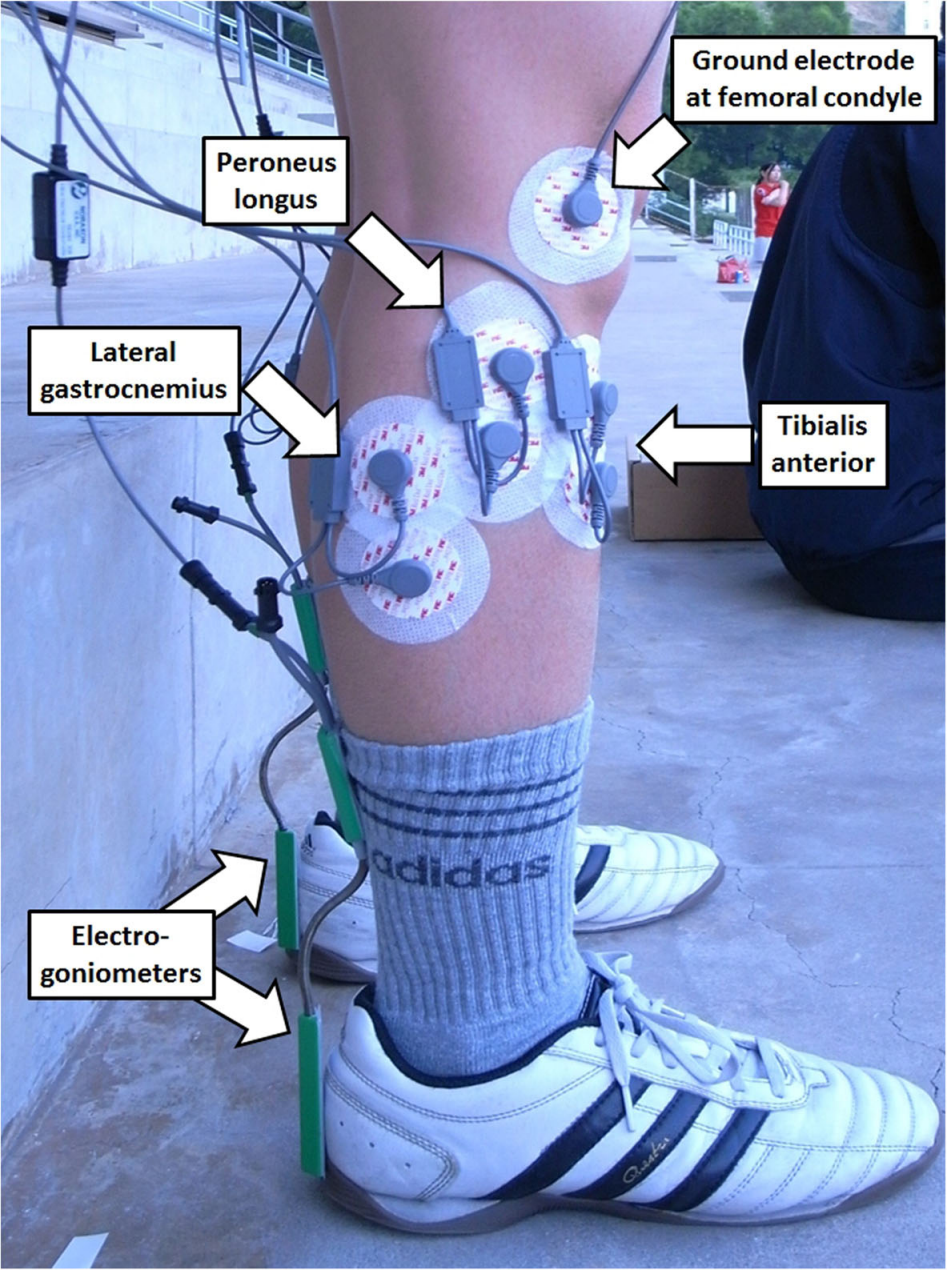
The locations of electrodes for collecting electromyography signals, and the electrogoniometers for collection ankle inversion data

The ankle muscle reaction test was conducted at every 15-min interval, i.e. at 0, 15, 30, 45, 60, 75, 90, and 105 min. The subject stepped on a pair of ankle sprain simulators which elicited an ankle muscle reaction to resist sudden simulated ankle inversion perturbation [27]. Three pairs of surface electrodes (3 M Red Dot Electrodes, diameter = 6 cm, inter-electrode distance = 2 cm, USA) were attached on the skin surface of the peroneus longus, tibialis anterior and lateral gastrocnemius muscles. The electromyography (EMG) signals were collected via an EMG system with 1000 Hz sampling rate (TeleMyo 2400 T G2, Noraxon Inc., USA), as shown in Fig. 2. The muscle positions were identified by a method suggested in an EMG manual [28]. A reference electrode was attached to the lateral femoral condyle. Subjects were instructed to stand on the platforms as they usually do on hard ground, with weight equally distributed on both limbs and without additional lower limb muscle contraction as monitored by the EMG system. Sudden simulated ankle inversion perturbations were introduced to the dominant and non-dominant limb in random sequence, until three trials on each limb were collected. Dominant limb was defined as the preferred limb to kick a ball as verbally reported by the participant. Two electrogoniometers (SG110, Biometrics Ltd., UK) were attached to the posterior shanks and heels to identify the time of the start of inversion as when the inversion angle changed by 0.1 degrees. The time of onset of the EMG signal at each muscle was determined by a sudden signal increase which exceeded 5% of the maximum signal value of each channel. The time between the start of ankle inversion and the onset of EMG signal was the reaction time of each muscle. Each test took approximately 2 min, and the subject resumed the exercise protocol immediately after. The average value of the reaction time from the three trials was used in the analysis.

### Statistical analysis

Repeated measures one-way multivariate analysis of variance (MANOVA) was conducted on the dependent variables over time. If a significant time effect was found, a post-hoc Bonferroni test was conducted to evaluate if the reaction time at each time point significantly differed from that at baseline. Statistical significance was set at *p* < 0.05 level.

## Results

**Fig. 3:**
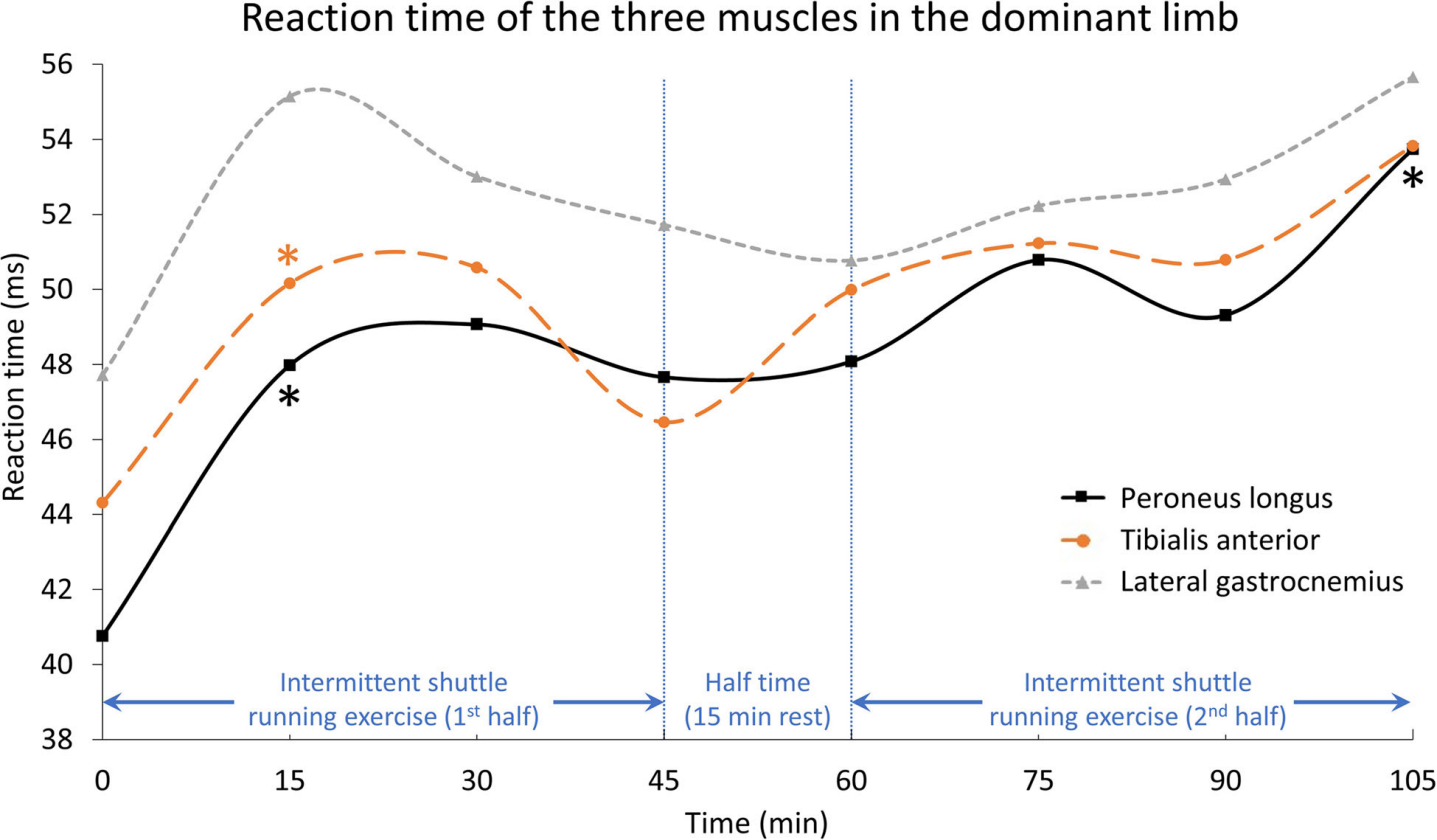
Reaction time of the three muscles in the dominant limb

**Fig. 4:**
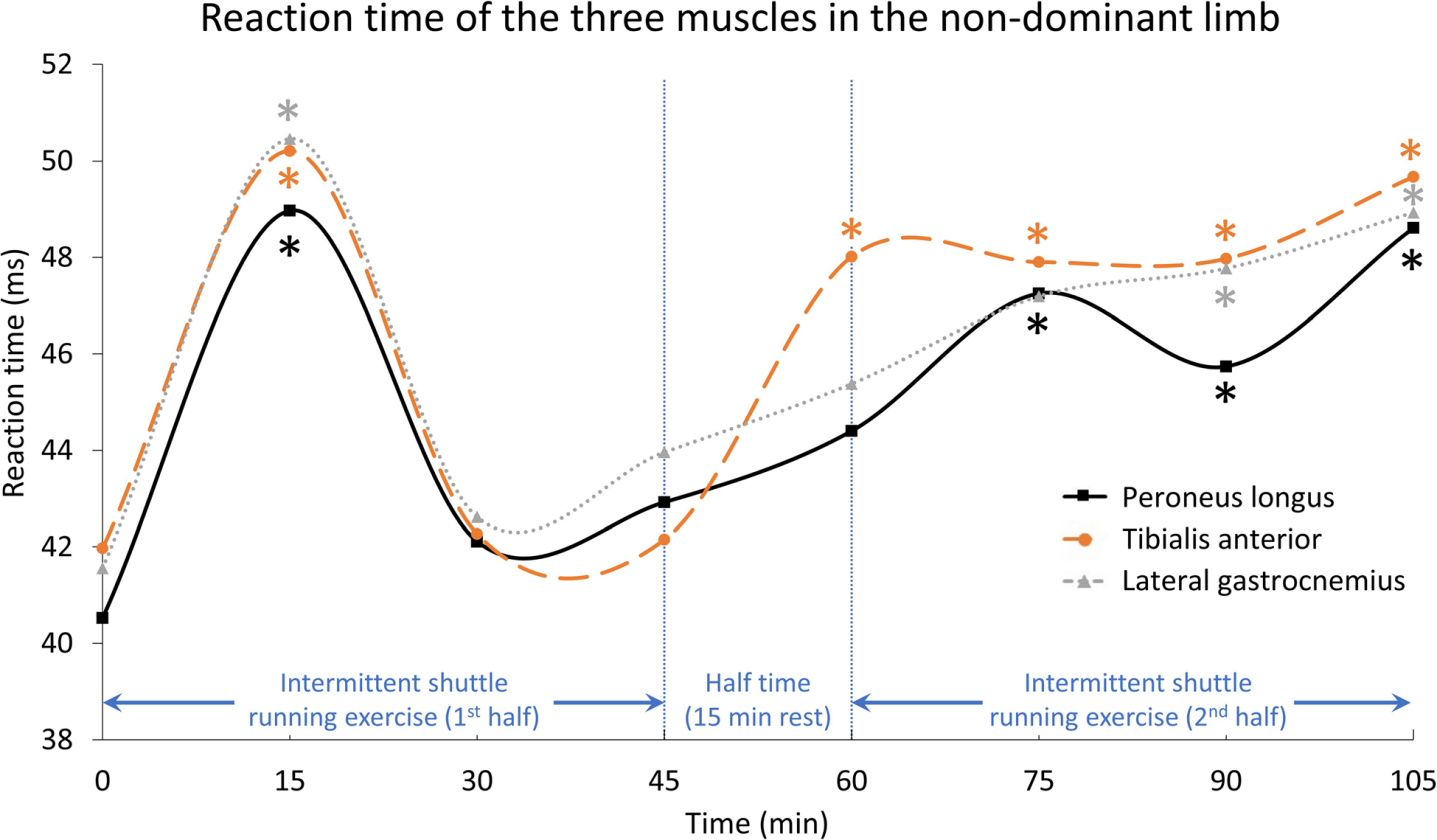
Reaction time of the three muscles in the non-dominant limb

Table 1 shows the demographic data of the participants. Table 2, Figs. 3 and 4 show the reaction time data for each of the three muscles for the dominant and non-dominant limbs at each measurement point. MANOVA suggested a significant time effect for the muscle reaction time (Wilks’ Lambda = 0.374, F = 10.028, *p* = 0.019). Post-hoc tests indicated that the reaction times differed in both the dominant and non-dominant limbs compared with baseline. For the dominant limb, a longer reaction time was found after 15 min on the peroneus longus and tibialis anterior. For the non-dominant limb, a longer reaction time was also found after 15 min for all three muscles, and subsequently at 60 min on the tibialis anterior, at 75 min on the peroneus longus, and at 90 min on the lateral gastrocnemius. These longer reaction times of the muscles of the non-dominant leg lasted until the end of the protocol (105 min).

**Table 1:** Demographic data of the participants (*N* = 7)

Parameter	Mean ± Standard Deviation
Age	25.7 ± 1.3 years
Height	1.6 ± 0.1 m
Body mass	51.1 ± 4.3 kg

**Table 2:** Reaction times of each of the three muscles in the dominant and non-dominant limbs at different time points (standard deviation in bracket)

	Peroneus Longus				Tibialis anterior				Lateral gastrocnemius			
	Dominant		Non-dominant		Dominant		Non-dominant		Dominant		Non-dominant	
Time (min)	Reaction (ms)	Sig	Reaction (ms)	Sig	Reaction (ms)	Sig	Reaction (ms)	Sig	Reaction (ms)	Sig	Reaction (ms)	Sig
0	40.76 (10.90)	–	40.52 (2.95)	–	44.31 (12.05)	–	41.97 (2.72)	–	47.72 (11.35)	–	41.56 (4.06)	–
15	47.98 (7.32)	.012*	48.97 (9.02)	.043*	50.16 (10.37)	.004*	50.21 (9.13)	.030*	55.14 (7.42)	.087	50.46 (8.92)	.037*
30	49.07 (3.00)	.083	42.11 (8.86)	.701	50.58 (3.88)	.222	42.27 (9.10)	.941	53.01 (4.64)	.313	42.62 (9.36)	.833
45	47.66 (6.33)	.072	42.92 (7.06)	.495	46.46 (7.05)	.604	45.15 (6.02)	.322	51.72 (6.31)	.423	43.96 (6.41)	.468
60	48.08 (7.20)	.239	44.40 (6.23)	.162	49.98 (8.04)	.373	48.01 (7.20)	.034*	50.77 (7.93)	.598	45.38 (5.16)	.253
75	50.78 (4.14)	.077	47.25 (5.35)	.001*	51.23 (4.46)	.160	47.90 (5.92)	.006*	52.23 (5.45)	.332	47.19 (7.75)	.051
90	49.31 (5.98)	.196	45.74 (4.00)	.001*	50.78 (6.55)	.328	47.93 (6.15)	.012*	52.94 (7.02)	.387	47.77 (4.31)	.018*
105	53.74 (4.76)	.049*	48.61 (4.39)	.003*	53.83 (4.94)	.136	49.67 (6.62)	.017*	55.66 (7.65)	.200	48.93 (5.23)	.010*

*p < 0.05

## Discussion

The results of the current study suggested that peroneal muscle fatigue may happen after the first 15 min of a simulated functional prolonged football protocol, and may cause delayed reaction time of the ankle muscles in this group of amateur female football players. A recent systematic video analysis reported that 68% of anterior cruciate ligament injuries happened in the first half, and one-quarter of them in the first 15 min of a football match [29]. This is probably due to a potentially higher injury risk caused by muscle fatigue [30] and subsequent reduction in postural control [31]. The authors suggested that this was possibly due to the inadequate neuromuscular readiness of fresh, unfatigued players [29]. Although that study was on another type of injury, the findings concur with the present study that muscle fatigue might already occur in the first 15 min of a match. This leads to delayed peroneal muscle times, as well as a greater risk of anterior cruciate ligament injuries. We observed that the reaction times plateaued after the first 15 min in the dominant limb (Fig. 3), before returning to nearer baseline at the 30th and 60th minutes. Subsequently, reaction times were slower again after the 75th minute, primarily in the non-dominant limb (Fig. 4). The reaction times of the studied muscles started from 40.5–47.7 ms and increased to 48.6–55.7 ms at the end of the simulated protocol (105 min). Although these reaction times were still within the range found for healthy people, of 55–80 ms [32], the longer time in these athletes may be too great to react to a quick ankle sprain motion that happens within 50 ms [16]. The slower reaction time may thus impair functional ankle joint stability and result in more ankle injuries during the later stages of a football match [33].

Apart from the dominant lateral gastrocnemius, one interesting observation was the significantly increased reaction time in all muscles, from 40.5–44.3 ms to 48.0–50.5 ms, after the first 15 min. The reaction time dropped back to 42.1–50.6 ms at 30 min but increased again at the end of the exercise protocol. Such acute delays in reaction time were also reported in a recent study with 10 male football players running for 45 min on a treadmill, and who had their reaction time to accommodate balance perturbation recorded every 7.5 min [34]. The reaction time in response to inversion perturbation, compared to that at the start (63 ms), did not differ at 7.5 and 15.0 min, but raised significantly to 84–90 ms at 22.5, 30.0, and 37.5 min, before returning to baseline, with no difference recorded at 45 min. While the results of this study could not provide additional information to explain this finding, we believe that this acute delay in muscle reaction time may be a result of central fatigue, or central activation deficit, which has been demonstrated in prolonged running exercise in previous studies [35]. Unfortunately, to date, it is still very difficult to have a reliable quantitative method to investigate central fatigue. As discussed earlier, a recent study showed a quarter of anterior cruciate ligament injuries are recorded in the first 15 min of a football match [29]. This may imply that the readiness of a football player in term of muscle reaction and dynamic joint stability were not yet optimal after the conventional warm-up. Modification of warm-up protocol could be done by introducing new elements such as post-activation potentiation which was found to boost the performance of jumping and cutting movements in male footballers [36]. By introducing a warm-up exercise of moderate intensity (e.g. 80%-1RM back squat) just before a football match, it might induce greater post-activation potentiation. This could improve the warm-up effect on the muscle activity and dynamic joint stability, but more research is needed.

Another interesting finding was the occurrence of delayed muscle reaction time in the non-dominant but not the dominant limb. De Luca and colleagues [37] conducted a myoelectric investigation on the fatigue at the first dorsal interosseous muscle and found that muscle fatigue happened faster in the non-dominant hand of right-handed individuals, but not in left-handed subjects. Farina and colleagues [38] also reported that the upper trapezius muscle at the non-dominant side was less fatigable in an isometric contraction test. To date, no studies have investigated the fatigue or reaction time of lower limb muscles during prolonged exercise.

There have been numerous attempts to deliver exercise training interventions to prevent inversion type ankle sprain injuries. Linford and colleagues [39] reported that a 6-week neuromuscular training programme was effective in reducing the reaction time of the peroneus longus muscles in a group of 26 healthy subjects. Similarly, Eils and Rosenbaum [40] reported that a 6-week multi-station proprioceptive exercise programme improved muscle reaction time, as well as joint position sense and postural sway. Interestingly, some other studies with proprioceptive and balance training as an intervention showed no reduction in muscle reaction time [41], but a significant effect in reducing ankle sprain incidents [42]. A previous study may give clues to this phenomenon. Sheth and colleagues [43] delivered an 8-week ankle disk training to 10 subjects and compared the contract time and pattern of four muscles – anterior tibialis, posterior tibialis, peroneus longus, and flexor digitorium longus. Although an expected decrease of reaction time did not happen, the contraction sequence changed to favour the correction of excessive ankle inversion. Before training, the four muscles tended to contract at the same time for a stiff ankle joint to accommodate excessive ankle inversion as initiated by a trapdoor platform. After training, the anterior and posterior tibialis contracted at a later time. These muscles are ankle invertors, so when they are inactive, they allow more eversion moment as generated by the ankle evertor, i.e. the peroneus longus. We believe that, if feasible, peroneal muscle endurance should be assessed in the preseason period [44], to understand the physical qualities and functional test performance of the football players and to design and guide injury prevention strategies.

## Conclusions

In this study, a delayed reaction time of the ankle muscles was found after the first 15 min and towards the end of a simulated prolonged football protocol. The ankle muscle reaction time increased to values greater than the time taken for an ankle sprain motion (< 50 ms). Such an increase may impair ankle joint stability, potentially resulting in more ankle injuries during the later stages of a football match. Acute delayed muscle reaction was observed in the first 15 min, which may be a result of central fatigue or central activation deficit. Future injury prevention strategies should also try to focus on tackling the delayed ankle muscle reaction time in the acute phase (the first 15 min), in addition to the latter minutes in the second half.

## Data Availability

The datasets used and/or analysed during the current study are available from the corresponding author on reasonable request.
